# MetaMind: A multi-agent transformer-driven framework for automated network meta-analyses

**DOI:** 10.1371/journal.pone.0342895

**Published:** 2026-02-13

**Authors:** Achilleas Livieratos, Maria Kudela, Yuxi Zhao, All-shine Chen, Xin Luo, Junjing Lin, Di Zhang, Sai Dharmarajan, Sotirios Tsiodras, Vivek Rudrapatna, Margaret Gamalo

**Affiliations:** 1 Independent Researcher, Athens, Greece; 2 Pfizer Inc., Collegeville, Pennsylvania, United States of America; 3 Takeda Pharmaceuticals U.S.A., Inc., Cambridge, Massachusetts, United States of America; 4 Teva Pharmaceuticals USA, Inc., West Chester, Pennsylvania, United States of America; 5 Sarepta Therapeutics, Inc., Cambridge, Massachusetts, United States of America; 6 4th Department of Internal Medicine, Attikon University Hospital, Athens, Greece; 7 Division of Gastroenterology and Hepatology, Department of Medicine, University of California, San Francisco, California, United States of America; Iowa State University, UNITED STATES OF AMERICA

## Abstract

**Background:**

Network meta-analysis (NMA) can compare several interventions at once by combining head-to-head and indirect trial evidence. However, identifying, extracting, and modelling these often takes months, delaying updates in many therapeutic areas.

**Objective:**

To develop and validate *MetaMind*, an end-to-end, transformer-driven framework that automates NMA processes—including study retrieval, structured data extraction, and meta-analysis execution—while minimizing human input.

**Methods:**

*MetaMind* integrates Promptriever, a fine-tuned retrieval model, to semantically retrieve high-impact clinical trials from PubMed; a multi-agent LLM architecture--Mixture of Agents (MoA)-- pipeline to extract PICO-structured (Population, Intervention, Comparison, Outcome) endpoints; and GPT-4o–generated Python and R scripts to perform Bayesian random-effects NMA and other NMA designs within a unified workflow. Validation was conducted by comparing *MetaMind’s* outputs against manually performed NMAs in ulcerative colitis (UC) and Crohn’s disease (CD).

**Results:**

Promptriever outperformed baseline SentenceTransformer with higher similarity scores (0.7403 vs. 0.7049 for UC; 0.7142 vs. 0.7049 for CD) and narrower relevance ranges. Promptriever performance achieved 82.1% recall, 91.1% precision and an F1 score of 86.4% when compared to a previously published NMA. *MetaMind* achieved 100% accuracy on a limited set of remission endpoints regarding PICO (Population, Intervention, Comparator, Outcome) element extraction and produced comparative effect estimates and credible intervals closely matching manual analyses.

**Conclusions:**

In our validation studies, *MetaMind* reduced the end-to-end NMA process to less than a week, compared with the several months typically needed for manual workflows, while preserving statistical rigor. This suggests its potential for future scaling of evidence synthesis to additional therapeutic areas.

## Introduction

NMA is a cornerstone of evidence-based medicine (EBM), providing a robust framework for comparing multiple interventions simultaneously. NMAs combine results from direct head-to-head trials and indirect comparisons, allowing estimation of treatment effects even when not all therapies have been compared in a single study. However, conducting NMAs is a resource-intensive process that requires significant manual effort, including study identification, data extraction, statistical modelling, and evidence synthesis [1–4]. These demands require substantial lead time to be effective and often create operational bottlenecks, which in turn limit the timely availability of up-to-date network meta-analyses across many therapeutic areas. As a result, evidence gaps persist, leading to suboptimal treatment decisions for patients and hindering advancements in precision medicine. Because NMAs demand both statistical expertise and manual screening of hundreds of studies, many therapeutic areas — including inflammatory bowel disease — have gone years between updates. The challenges associated with NMA workflows—including data heterogeneity (from a population and research design/operation), bias assessment, and the need for consistent data structuring—further complicate the process [1–4].

Artificial intelligence (AI) has the potential to transform NMA workflows by automating many of the manual processes involved, from study identification to data extraction, analysis, and interpretation. Advances in large language models (LLMs) and transformer-based retrieval systems have demonstrated remarkable capabilities in natural language processing, medical literature synthesis, and structured data extraction [5–7]. AI-driven automation could alleviate the burdens associated with traditional NMA workflows, making the process faster, more accurate, and scalable [1–4]. However, despite these advancements, there is currently no fully integrated, end-to-end software solution for conducting NMAs across therapy areas in a streamlined, reproducible, and flexible manner [1–4]. Most existing AI-assisted approaches focus on isolated tasks, such as data extraction or statistical modelling, without offering a holistic pipeline that seamlessly integrates all stages of the NMA process.

Here we describe and validate *MetaMind*, a new method for automating NMAs steps with minimal human input. This approach integrates a transformer-based retrieval system, Promptriever, with a multi-agent large language model framework to automate study retrieval, data extraction, and meta-analysis execution. This workflow removes several high-burden steps from conventional NMA processes, without loss of accuracy, and can be applied to multiple disease areas without substantial re-engineering. We illustrate and validate its application in a comparative efficacy study for Ulcerative colitis and Crohn’s disease, demonstrating its robustness, adaptability, and clinical utility. By automating key stages of the NMA process, our approach reduces the time required from months to days, making it feasible to apply similar workflows in other disease areas.

While prior work has shown that structured data can be extracted from published NMAs for downstream reanalysis, such approaches are limited to post hoc extraction from already completed analyses [1]. These methods do not address the upstream and more labor-intensive steps of the NMA lifecycle—namely, primary RCT retrieval. In contrast, *MetaMind* introduces a fully integrated, end-to-end framework built entirely in Python, which combines retrieval (Promptriever), layered MoA extraction, and dynamic Bayesian NMA script generation and execution via GPT-4o. This is the first framework, to our knowledge, to unify the entire NMA pipeline from efvidence identification through final analysis using MoA in a reproducible, extensible manner—representing a substantial methodological advancement beyond isolated automation components. *MetaMind* automates the core computational stages of network meta-analysis—study retrieval, data extraction, and statistical model execution—within a unified pipeline. While not fully autonomous in areas such as feasibility assessment or model validation, it provides an extensible framework for end-to-end automation of the technical workflow, substantially reducing manual workload and turnaround time.

## Methods

This study reports the development and validation of a new method, *MetaMind*, for automating network meta-analyses of clinical studies. To validate *MetaMind*, we used it to estimate the comparative efficacy of therapies in Ulcerative colitis and Crohn’s disease and compared our results with the results of manually performed NMAs in these therapeutic areas. Performance was evaluated against multiple manually curated reference NMAs, including independent Ulcerative Colitis and Crohn’s disease networks, as well as manually implemented R-based analyses.

### Methodological components and implementation

**Fig 1:**
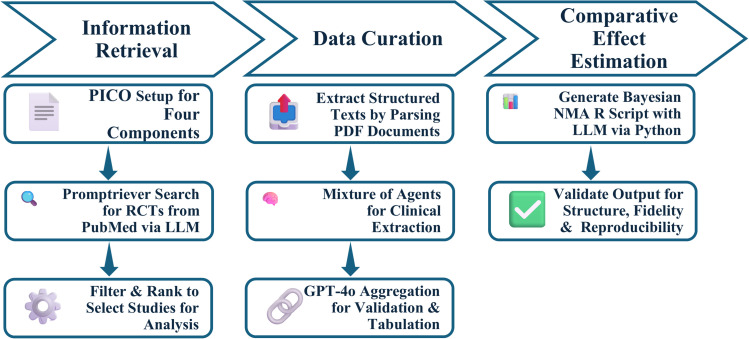
Workflow Schematic.

The overview of our method called *MetaMind* is described in Fig 1, and involved chaining together several modules including information retrieval, data curation, and comparative effect estimation. This study aimed to develop and evaluated an AI-driven framework for structured clinical evidence synthesis, applying advanced retrieval and extraction methodologies to comparative efficacy analysis in moderate-to-severe inflammatory bowel disease (IBD). Specifically, the approach was designed to extract and analyze clinical trial data for UC and CD using promptable PICO metrics to ensure targeted retrieval of relevant studies. To achieve this, Promptriever was employed to search and retrieve high-impact clinical trials from PubMed, focusing on studies assessing biologic and small-molecule therapies compared to placebo or active comparators. In this work, we pre-selected the relevant studies for downstream analysis for ease of comparability and validation with the manual Bayesian Evidence Generation. The MoA framework was then applied to extract key clinical endpoints, including baseline and final remission rates, sample sizes, and confidence intervals. This structured extraction, which was FLASK (Fine-grained Language model evaluation based on Alignment Skill Sets) evaluated, enabled a comprehensive evaluation of treatment efficacy across heterogeneous study designs, facilitating a scalable and potentially generalizable methodology for automated evidence synthesis in IBD research.

To operationalize PICO-aligned retrieval and extraction, we used structured, model-ready prompts tailored to each stage of the pipeline:

Retrieval via Promptriever was guided by user-defined PICO elements embedded into a query-instruction format. For example, a prompt submitted to the model might read:

*“Query: ulcerative colitis AND placebo AND (mirikizumab OR upadacitinib OR filgotinib OR ustekinumab OR etrasimod OR tofacitinib). A relevant document would describe clinical trials where patients were tested against placebo and the document includes efficacy results for the named treatments.”*

This query format supports retrieval based on the PICO framework—specifying patient group, intervention, comparator, and outcome. Extraction via MoA system used standardized prompts across models like Meta-LLaMA-3, Mistral, and Qwen2 to extract structured clinical trial data. A representative example prompt is:

*“Extract baseline and final clinical remission values for each treatment group, including confidence intervals (CIs) or standard deviations (SDs) where available. Report weekly remission rates at Weeks 4, 8, and 12. Include sample sizes per group. If any values are missing, clearly indicate and suggest plausible methods for estimation. Format output as a structured table for network meta-analysis.”*

These prompts enabled reliable, cross-validated extraction of quantitative endpoints from free-text PDF trial reports. The detailed workflow is provided in Supplementary Figures 1 & 2 in S1 File.

The NMA generated through this approach was fully scripted and implemented using AI-driven automation, with GPT-4o producing the analytical script in Python. To validate the accuracy and robustness of the AI-generated NMA, results were benchmarked against a manual Bayesian Evidence Generation, conducted using R-based statistical methodologies widely accepted in evidence synthesis. This dual-implementation strategy allowed for a direct comparison between AI-assisted and traditional statistical approaches, ensuring methodological rigor and alignment with best practices in clinical research and decision-making (Fig 1).

*Metamind* workflow was then compared to a published manual NMA, to further confirm findings between the two approaches across all 3 stages of information retrieval, data curation, and comparative effect estimation [8].

### Retrieving NMA-relevant articles using promptriever

Promptriever is implemented in PyTorch atop a Llama-2 base Transformer with a Parameter-Efficient Fine-Tuning (PEFT) adapter. We then fixed both model and tokenizer to a maximum sequence length of 512 tokens (with padding to multiples of eight to maximize batch efficiency). We did not perform any additional training or fine-tuning of Promptriever; instead, we used the publicly released pre-trained model [6].

At inference time, user prompts are grouped into batches of up to four which tokenizes them with truncation and pad-to-max-length. We extract the first token embedding for each sequence—yielding a (B, 256) tensor—apply ℓ₂-normalization across the feature dimension, and concatenate results into an (N, 256) NumPy array for all inputs.

These normalized embeddings are then compared against our library of template embeddings stored in a FAISS index. We compute inner-product similarity scores between each query vector and every template, sort in descending order, and retain the top candidates (whereby the retrieval parameter default is 100). To capture fine-grained contextual fit, each retrieved template is concatenated with the original prompt and fed to a lightweight 4-layer MLP re-ranker (512 units, ReLU activations, dropout 0.1). This network outputs a scalar relevance score for each pair.

We benchmark against the pre-trained all-mpnet-base-v2 SentenceTransformer (224 M parameters). This model generates 768-dim embeddings for both user prompts and templates. We follow the identical retrieval pipeline: compute cosine similarities over all template embeddings, sort and retain the top results (default: 100), and re-rank via the same 4-layer MLP described above. All other are kept constant to ensure direct comparability.

To facilitate a controlled validation against previously published manual NMAs, we limited the retrieval scope to a maximum of 10 high-impact articles per disease area, specifically from *The Lancet* and *NEJM*. This selection allowed for benchmarking model performance against high confidence, widely cited RCTs with standardized reporting formats. However, this constraint reflects a design choice for comparability—not a limitation of *MetaMind* itself. As shown in S1 File, when applied without restrictions, Promptriever unrestricted application was performed against a comprehensive manual review. This demonstrates *MetaMind’s* capacity to scale beyond artificial benchmarks, enabling broader and more current real-world literature synthesis. We examined recall, precision, and F1 to evaluate our findings.

Our implementation reuses and adapts components from the Weller et al. publication, with modifications for domain-specific retrieval (Supplementary Figure 1 in S1 File) and integration into the NMA pipeline [6].

### Text extraction and preprocessing

The next step of this methodology focused on extracting text from PDF documents, leveraging the Fitz library (PyMuPDF) to systematically gather all available textual data, such as abstracts and clinical study details. Many PubMed records are machine-readable (XML or JSON), but full-text availability is limited by subscription access, publisher restrictions, and inconsistent file formats. PDFs present unique challenges, including inconsistent structure, embedded images, and encoding issues, making them a rigorous test case for our Mixture of Agents approach. Tables formatted as text were successfully processed; however, images, scanned tables, and graphical elements (e.g., plots) were not used in this version of the pipeline. Thus, results rely on data being presented in machine-readable textual formats. Future extensions may incorporate optical character recognition-based extraction for image-based tables or scanned documents.

By prioritizing PDF extractions, this methodology demonstrated the robustness and adaptability of our system in handling real-world document retrieval scenarios. A structured list was created, where each entry corresponded to a single PDF document, and exception handling mechanisms were implemented to address issues such as unreadable files or unsupported formats. This step was critical in preparing raw text data for subsequent semantic processing by LLMs, ensuring high-quality information retrieval even from complex, unstructured sources.

### Prompt engineering and layered aggregation

The methodology employed a MoA approach, utilizing multiple LLMs across distinct inference layers to optimize clinical parameter extraction from both structured PubMed data and unstructured full-text PDFs. In the first inference layer, models including Meta-Llama-3.1-8B-Instruct-Turbo, Mistral-7B-Instruct-v0.3, and Qwen2-72B-Instruct were tasked with extracting baseline and final clinical values, standard deviations, confidence intervals, and sample sizes. To improve accuracy and consistency, the second and third inference layers employed GPT-4o, which aggregated model outputs, cross-validated extracted clinical parameters, recalculated missing values when necessary, and structured the results into a standardized format suitable for NMA. This layered approach enhanced robustness, particularly in complex PDF extractions, where inconsistent formatting, missing values, and embedded numerical data posed significant challenges. The methodology applied a recursive approach where the GPT-4o model in each layer refined the aggregated outputs from the previous iteration (Supplementary Figure 2 in S1 File). The full MoA implementation, including agent prompts, aggregation logic, and decision rules, is provided in the Appendix. This design is model- and disease-agnostic and does not rely on condition-specific heuristics.

### Final synthesis

In the final stage, the responses from the aggregation layers were synthesized into a comprehensive output using GPT-4o. In this final stage, the pipeline used GPT-4o’s streaming mode to generate outputs progressively from structured prompts. All prompts were fixed in advance and applied uniformly across documents. This streaming functionality refers solely to the LLM response interface and does not imply iterative fine-tuning, editing, or adaptive prompting. Extracted data were compared post hoc to manually curated NMA reference sets for evaluation.

### PICO

The output of this methodology included a manual evaluation of PICO elements, assessing the accuracy (faithful reproduction) and completeness of the generated summaries in relation to the reference text [2]. Rather than relying on similarity scores, which proved insufficient due to the highly detailed and comprehensive nature of the model outputs, each PICO component was individually and manually reviewed to ensure factual correctness, alignment with the reference, and clinical relevance. A qualitative assessment approach was applied, ensuring that evaluations captured nuanced differences in medical evidence synthesis.

This manual approach is particularly valuable in clinical research and systematic reviews, where accurate representation of PICO elements is essential for high-quality evidence synthesis. By prioritizing expert-driven assessment over purely computational similarity measures, the methodology ensures a rigorous and context-aware evaluation of LLM-generated summaries, setting a higher standard for automated text summarization systems in medical applications.

### FLASK

This methodology employed a multi-dimensional evaluation framework using the FLASK criteria, which assessed textual outputs based on correctness, factuality, efficiency, commonsense, comprehension, insightfulness, completeness, metacognition, readability, conciseness, and harmlessness. The evaluation process was fully automated, leveraging OpenAI’s GPT-4o to systematically score and analyse responses [5,9]. First, the reference text was extracted from clinical study PDFs, ensuring a structured comparison between the original document and the LLM-generated response. GPT-4o was then prompted with a predefined evaluation rubric, requesting numerical scores (1–5) for each FLASK criterion, along with a brief justification for each score. By integrating automated scoring and structured evaluation, this framework ensured objective, reproducible, and high-fidelity assessments of LLM-generated clinical summaries (Supplementary Figure 3 in S1 File).

In addition to automated evaluation, we performed manual spot checks on a random sample of outputs to ensure alignment between LLM-assigned FLASK scores and human clinical interpretation. This hybrid approach helped verify that model judgments were consistent with domain-specific expectations. LLM-based scoring can scale evaluation across large datasets, but the models may reflect biases present in their training data. To mitigate this, we used fixed rubrics and instructed the model to justify each score. This approach, along with manual spot validation, was designed to reduce variability and surface potential inconsistencies in scoring across different documents.

### Automation of NMA generation

We used GPT-4o to write analytical code in R, using a Python-integrated workflow, to analyze the assembled dataset using brms, a validated and widely used software package for estimating comparative effects using Bayesian statistics (Supplementary Figure 4 in S1 File). This study employed an API-driven approach to dynamically generate and execute the code for Bayesian meta-analysis using GPT-4o [1]. Structured pseudocode instructions were provided to GPT-4o to generate the script that defines and analyses a Bayesian random-effects model. The model evaluated relative treatment effects with a binomial likelihood and logit link function, incorporating random treatment effects at the study level and specified priors. This script was executed within Python using the subprocess module, allowing seamless integration with the remaining Python-based workflow. AI-generated code in *MetaMind* serves as a scripting mechanism to faithfully reproduce a predefined, standard network meta-analysis workflow rather than to autonomously design or optimize statistical models. The underlying statistical approach was specified a priori and corresponds to established Bayesian NMA implementations. The role of the language model was limited to generating executable R scripts that implement this predefined analysis pipeline.

More specifically, the NMA code itself was written in R by GPT-4o using structured pseudocode instructions. The workflow runs primarily in Python, which calls an R-based Bayesian model through dynamically generated scripts executed via the subprocess module. This hybrid setup was chosen to preserve the statistical rigor of established R-based packages (e.g., brms), while enabling LLM-assisted automation and integration using Python.

To assess the statistical equivalence of AI-generated analysis code, results produced by GPT-4o–generated scripts were compared against manually implemented and vetted reference analyses using the same datasets and model specifications. Across these comparisons, treatment effect estimates, credible intervals, heterogeneity parameters, and treatment rankings were numerically equivalent, with no discrepancies in clinical conclusions observed. These checks confirm that AI-generated code faithfully reproduces standard Bayesian network meta-analysis workflows when executed under equivalent assumptions.

The generated script includes model definitions, priors, convergence diagnostics, and summary tables, and is available under Supplementary Materials in S1 File.

### Bayesian evidence generation

The Bayesian analysis workflow was implemented using a Python-integrated workflow to evaluate treatment effects through a binomial-logit regression framework. A Bayesian random-effects model was defined with parameters for treatment-specific effects and between-study heterogeneity, using appropriately specified priors. The log-odds were estimated by modelling response counts over total sample size, incorporating treatment as a fixed effect and random treatment effects at the study level. The model output included posterior summaries with credible intervals, Rhat values for convergence diagnostics, and an estimate of between-study heterogeneity (tau). Between-study heterogeneity is naturally quantified by the posterior distribution of τ; I², which is derived from frequentist statistics, was therefore not the primary heterogeneity measure reported.

The entire analysis was executed in R-integrated Python workflow via the subprocess module, enabling seamless automation and reproducibility of Bayesian meta-analysis (Supplementary Figure 4 in S1 File).

In our analysis we utilized an approach within the Bayesian framework extension, which offers certain advantages like user-friendliness and familiarity over the more common NMA approach that examines treatment contrasts [10–12]. The treatment contrast method is extensively employed in health technology evaluations, such as those conducted by the UK’s National Institute for Health and Care Excellence (NICE). The NICE decision support unit has provided methodological guidelines on the practical application of this method [13].

### Manual R-based NMA validation

To validate the results the manual implementation was performed for the set of selected publications [14–23]. Standard process was followed starting with comprehensive literature search and systematic review of selected studies. To enable fair comparison between manual and automated selection, we focused on selected studies. We extracted the data on study design, interventions, and outcomes. The results derived from our manual network meta-analysis closely mirrored those obtained via our automated pipeline approach, indicating a high degree of concordance between two approaches and affirming the robustness and reliability of the automated procedure (Supplementary Figures 5 & 6 in S1 File). In future endeavours, we plan to incorporate elements such as model evaluation, assessment of goodness-of-fit for the selected publications, and verification of underlying assumptions in the end-to-end pipeline [24,25].

### Workflow

This entire methodology was implemented in Python, with R executed within this ecosystem, eliminating the need to rely exclusively on R or other statistical programming languages traditionally associated with NMA (Supplementary Figures 1, 2, 4, 9 in S1 File) [1,7]. This approach retained the strengths of R for Bayesian modelling while leveraging Python’s integration capabilities for automation, machine learning, and natural language processing. To ensure *MetaMind* meets both rigor and speed requirements, we benchmarked its entire workflow runtime against typical manual NMA workflows.

### Framework for addressing study heterogeneity

Potential sources of clinical and methodological heterogeneity—such as dosing regimens, patient population characteristics, trial duration, and prior treatment exposure—were explicitly extracted as structured fields by MoA. In the current study, these variables were not incorporated as effect modifiers in the network meta-analysis model; however, their structured extraction ensures that heterogeneity sources are transparent rather than implicitly ignored.

*MetaMind* addresses study heterogeneity through two key mechanisms: (1) the MoA framework extracts detailed trial-level data—including baseline characteristics (e.g., disease severity, prior treatment exposure, sample size, age distributions)—into a structured tabular format, and (2) the final NMA stage models between-study heterogeneity explicitly using a random-effects Bayesian model, which accounts for variance across trials. While the current pipeline does not yet implement automated subgroup adjustment or meta-regression, the structured data output allows users to identify population differences and refine inclusion or stratification rules as needed. This modular design enhances interpretability and consistency across heterogeneous study sources.

For studies with incomplete or heterogeneous reporting (e.g., missing standard deviations or alternative summary statistics), supplementary prompts were used solely to flag missing information and to enumerate commonly used statistical imputation or transformation approaches (e.g., deriving standard deviations from confidence intervals or interquartile ranges). Importantly, these prompts did not autonomously apply statistical corrections or alter trial data used in the network meta-analysis. All such cases required explicit human review, and only standard, guideline-consistent methods were applied prior to analysis.

### Implementation and reproducibility

All experiments were conducted using a consistent and explicitly specified LLM version throughout the study. Specifically, all LLM-driven components—including trial extraction, aggregation, and code generation—used GPT-4 (denoted as GPT-4o) without version switching or adaptive model selection. This avoided variability introduced by model updates. The temperature parameter was set to zero for critically challenging sections, ensuring deterministic token generation. Wherever supported by the underlying frameworks, random seeds were fixed to further reduce nondeterminism. Wherever repeated executions were performed, we observed no meaningful variability.

The full *MetaMind* pipeline implementation, including retrieval, extraction, aggregation, and analysis scripts, is provided in the Appendix. Due to reliance on proprietary LLM APIs, the pipeline is not released as a standalone executable repository; however, all core logic, prompts, and workflow components necessary for reproduction and adaptation are fully documented.

### Automated code generation and execution

AI-generated code was executed in a secure, sandboxed computing environment with no external network access, preventing unintended data exfiltration. The execution environment was restricted to standard statistical libraries required for Bayesian network meta-analysis (e.g., brms). Second, all generated code was inspected by the authors prior to execution to verify that it performed only the intended analytical tasks. This inspection step ensured that the code adhered to established statistical practices and did not contain unsafe or extraneous operations. Third, the outputs of AI-generated analyses were systematically validated. Model results—including effect estimates, credible intervals, and treatment rankings—were cross-checked against known benchmarks and manually implemented reference analyses on selected datasets. Standard convergence diagnostics were reviewed to confirm proper model convergence and numerical stability.

## Results

### *MetaMind* promptriever performance and output

We first examined, *MetaMind* performance in retrieving relevant articles. The performance of PEFT Promptriever and SentenceTransformer was evaluated based on their similarity scores for both UC and CD. The highest similarity score achieved by PEFT Promptriever was 0.7403, marginally outperforming SentenceTransformer’s 0.7049 (Table 1). Additionally, PEFT Promptriever demonstrated a narrower similarity range (0.0814) compared to SentenceTransformer (0.0959), indicating a tighter focus on relevant articles and higher specificity in aligning to nuanced query instructions. Similarly, on CD PEFT Promptretriever demonstrates more consistent performance with a narrower range (0.0353), focusing on high-relevance results (Table 2). A filter was applied to select only the top 10 most relevant articles.

**Table 1:** Performance Comparison Between Retrieval Models for Ulcerative Colitis.

Model	Highest similarity	Lowest similarity	Range
PEFT Promptriever	0.7403	0.6589	0.0814
SentenceTransformer	0.7049	0.6090	0.0959

**Table 2:** Performance Comparison Between Retrieval Models for Crohn’s Disease.

Model	Highest similarity	Lowest similarity	Range
PEFT Promptriever	0.7142	0.6789	0.0353
SentenceTransformer	0.7345	0.6368	0.0977

The top five articles on the induction and maintenance therapy for ulcerative colitis were filtered (Table 3). The study on Etrasimod achieved a similarity score of 0.7043. Upadacitinib scored 0.6998 and explored advanced therapies for moderate to severe ulcerative colitis These findings highlight the capability of PEFT Promptriever to retrieve high-quality, clinically relevant studies with nuanced semantic alignment.

**Table 3:** Promptriever Output-Key Ulcerative Colitis Articles Examples.

Title	Journal	Publication date	Similarity
Etrasimod as induction and maintenance therapy (ELEVATE)	Lancet (London, England)	2023-04-08	0.7043
Upadacitinib as induction and maintenance therapy	Lancet (London, England)	2022-06-04	0.6998
Filgotinib as induction and maintenance therapy (SELECTION)	Lancet (London, England)	2021-06-19	0.6928
Tofacitinib as induction and maintenance therapy	The New England Journal of Medicine	2017-05-04	0.6842
Ustekinumab as induction and maintenance therapy	The New England Journal of Medicine	2019-09-26	0.6589

A similar approach was taken for Crohn’s disease. The top four articles on the induction and maintenance therapy for Crohn’s disease were filtered (Table 4). The study on Risankizumab achieved the highest similarity score of 0.7142 and focused on clinical remission in moderate-to-severe Crohn’s disease.

**Table 4:** Promptriever Output-Key Crohn’s Disease Articles Examples.

Title	Journal	Publication date	Similarity
Induction therapy with risankizumab in moderate-to-severe Crohn’s disease	Lancet (London, England)	2017-04-29	0.7142
Efficacy and safety of mirikizumab in moderately-to-severely active Crohn’s disease	Lancet (London, England)	2024-12-14	0.7136
Risankizumab as induction therapy for Crohn’s disease (ADVANCE and MOTIVATE trials)	Lancet (London, England)	2022-05-28	0.7104
Clinical remission in Crohn’s disease patients treated with filgotinib (FITZROY study)	Lancet (London, England)	2017-01-21	0.6982

To validate Promptriever’s retrieval accuracy, we compared its results against a published NMA which included 28 relevant trials (Supplementary Figure 7 in S1 File). Of these, Promptriever successfully retrieved 23, resulting in a recall of 82.1%. All retrieved trials were relevant, yielding a precision of 91.1% and an F1 score of 86.4%. The 5 studies missed by Promptriever included 1 without a PubMed ID, which was not retrievable. These findings demonstrate that Promptriever delivers high-recall, high-precision semantic retrieval while also surfacing more recent and diverse studies than the manual reference set. Across reference NMAs, retrieval performance was consistent, with no qualitative differences observed between UC and CD. Moreover, as not every alternative Promtriever frameworks was explored, it is plausible that due to workflow flexibility these summary statistics can be further improved. Precision and recall were computed deterministically with respect to fixed retrieved trial sets. Because no sampling or resampling procedure was used and model inference was not involved in metric computation, these performance measures are reported as point.

### *MetaMind* MoA output

*MetaMind’s* MoA module was evaluated for its ability to extract structured clinical data from unstructured PDF trial reports. For selected trials in UC and CD, the system correctly extracted numerical endpoints (e.g., remission rates, confidence intervals, and sample sizes) across two timepoints per study, demonstrating successful mapping to NMA-ready tabular format (Table 5 & Table 6). These outputs were validated against manually curated references to assess extraction fidelity.

**Table 5:** Data Extraction for UC of Ustekinumab using MoA.

Treatment	Time point	Remission rate (%)	SE	95% CI	Sample size
Ustekinumab (130 mg)	Week 8	15.6	0.0207	11.5–19.7	321
Ustekinumab (6 mg/kg)	Week 8	15.5	0.0206	11.4–19.6	320
Placebo	Week 8	5.3	0.0125	2.8–7.8	319
Placebo	Week 44	24.0	0.0327	17.6–30.4	175
Ustekinumab (90 mg every 12 weeks)	Week 44	38.4	0.0371	31.1–45.6	172
Ustekinumab (90 mg every 8 weeks)	Week 44	43.8	0.0373	36.5–51.1	176

**Table 6:** Data Extraction for CD of Upadacitinib using MoA.

Trial	Treatment	Week 12 remission % (95% CI)	Week 52 remission %
U-EXCEL	Placebo	29.1 (22.4–35.8)	N/A
U-EXCEL	Upadacitinib 45 mg	49.5 (44.2–54.8)	N/A
U-EXCEED	Placebo	21.1 (14.9–27.2)	N/A
U-EXCEED	Upadacitinib 45 mg	38.9 (33.6–44.2)	N/A
U-ENDURE	Placebo	N/A	15.1
U-ENDURE	Upadacitinib 15 mg	N/A	37.3
U-ENDURE	Upadacitinib 30 mg	N/A	47.6

We assessed the accuracy of extracted PICO elements by manually reviewing outputs from a subset of UC and CD trials. For this limited evaluation (focused on remission-related endpoints), the MoA framework correctly extracted and aligned all targeted data fields, achieving 100% accuracy on a limited set of remission endpoints. Similar extraction performance accuracy was reported for other relevant data elements, including study design features, baseline patient characteristics, intervention and dosing information, and reported adverse events. Across reference NMAs, extraction performance was consistent, with no qualitative differences observed between UC and CD. Similarly, extraction performance was 100% when applied on papers from a recent, peer-reviewed NMA publication on UC [8]. However, we acknowledge that broader validation across multiple endpoints and trial designs is needed before generalizing this performance metric further.

FLASK variability was observed due to study-level differences in reported endpoints and formatting (Supplementary Figure 3 in S1 File). However, the use of universal prompts across all studies was sufficient to capture all required clinical information, even in the presence of structural variability (Supplementary Figure 2 in S1 File). This demonstrates the robustness of the prompt design, which enabled consistent extraction without the need for study-specific tailoring.

### *MetaMind* NMA output

**Fig 2:**
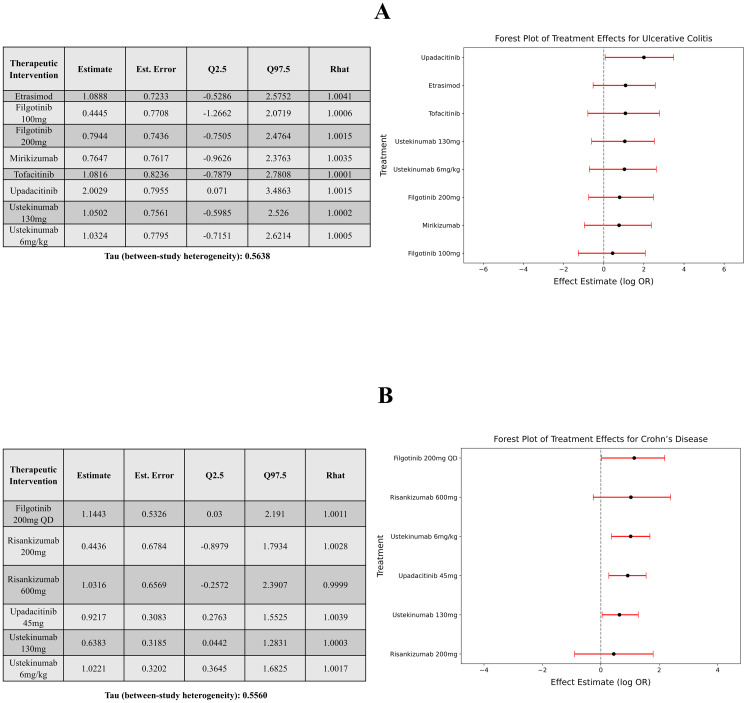
(a) UC NMA Summary & Forest Plot (b) CD NMA Summary & Forest Plot.

To validate the accuracy of *MetaMind’s* automated NMA pipeline, we conducted two complementary comparisons. First, we compared the AI-generated NMA results (Figs 2a and 2b) to manually performed analyses by the co-authors (Supplementary Figure 5 in S1 File) [14–23]. This internal benchmark showed high concordance in effect estimates, credible intervals, and treatment rankings, confirming the reliability of MetaMind’s outputs. For both disease networks, model convergence diagnostics indicated stable estimation across all parameters, with R-hat values approximately equal to 1.00 and effective sample sizes sufficiently large for all treatment effect estimates. For the ulcerative colitis network, the residual deviance (20.4 on 20 data points) was closely aligned with the number of observed data points, and the deviance information criterion (DIC) was 39.8, indicating adequate model fit under the specified random-effects structure. Similarly, for the Crohn’s disease network, the residual deviance (13.5 on 15 data points) demonstrated good agreement with observed data, and the DIC was 26.7, supporting satisfactory model fit. Network plots were constructed which were well-connected and contained evidence loops (Supplementary Figure 6 in S1 File). Second, we assessed alignment with a recent publication, a peer-reviewed NMA in ulcerative colitis (Supplementary Figures 7 and 8 in S1 File) [8,15,19,26–31]. This demonstrates consistent treatment effect patterns and overlap in identified trials, supporting external validity. Among treatments common to both *MetaMind* and the published NMA, relative treatment rankings and effect directions were concordant, with overlapping credible intervals for all comparable endpoints. These findings indicate that *MetaMind* can reproduce both internally generated and published NMA results.

Finally, *MetaMind* outputs can be generated within a week, while the entire workflow to be generated manually would require several months [32].

A stage-wise comparison of *MetaMind* against manual NMA workflows is summarized in Table 7, highlighting performance across retrieval accuracy, data extraction fidelity, statistical concordance, and execution time. Aggregate metrics are reported for clarity, as performance was consistent across benchmarks.

**Table 7:** Summary table of *MetaMind* performance across all stages.

Pipeline Stage	Metric	*MetaMind* Result
Retrieval	Recall (vs manual NMA)	82.1%
	Precision	91.1%
Data Extraction	PICO element accuracy	100%
NMA Concordance	Treatment overlap (UC/CD)	Comparable-No meaningful differences
	Tau, Rhat, CI diagnostics	Consistent with manual implementation
End-to-End Time	Total workflow duration	< 1 week (automated) vs ~ 3–4 months

## Discussion

The integration of advanced AI methodologies such as Promptriever and multi-agent architectures into NMA workflows signifies a shift in clinical evidence synthesis. The results underscore the efficiency of Promptriever’s PEFT-enabled retrieval system in surfacing highly relevant and nuanced PubMed studies, with evaluation metrics surpassing conventional retrieval models. By seamlessly coupling this retrieval mechanism with a multi-agent LLM framework, the pipeline ensures rigorous data extraction and synthesis, delivering structured and actionable insights with minimal human oversight. Observed similarity in effect estimates should be interpreted as evidence of faithful reproduction of standard Bayesian NMA workflows rather than proof of methodological uniqueness, particularly in settings where treatment effects are robust. By automating trial retrieval, data extraction, and model execution, the workflow cuts review times dramatically. In our case studies in ulcerative colitis and Crohn’s disease, it handled heterogeneous trial designs without major modifications.

This study focuses on improving the efficiency of NMA processes and developing a framework that can be reproduced and adapted in different settings.¹ ⁻ ²^,^⁵ Using open-source models in a multi-agent architecture allows tailoring to varied research needs.² We used prompt engineering to give LLMs precise instructions and context, which reduced manual oversight requirements [5,9]. Compared with a recent peer-reviewed manual NMA (Supplementary Figure 8 in S1 File), *MetaMind* matched traditional methods.

*MetaMind* is not a proprietary tool, but rather a reproducible research workflow built on open-source components and publicly available models. It is designed to be transparent, modifiable, and extensible for future researchers seeking to implement automated NMAs without relying on commercial software. *MetaMind* automates retrieval, extraction, and model fitting, but feasibility checks, eligibility screening, and model fit assessment were performed manually. These remain essential expert-guided tasks. Thus, our use of the term “end-to-end” refers specifically to the computational execution pipeline, not to full autonomy across all stages of NMA methodology

Overall, the workflow’s modular design, adaptability, and accuracy suggest its theoretical potential for wider use in automated evidence synthesis. The results from Promptriever’s PEFT-enabled retrieval in Step 1, the layered multi-agent extraction in Step 2, and the automated code generation and NMA execution in Step 3 exemplify a system designed to scale with evolving datasets, therapeutic areas, and analytical demands within a unified workflow.

## Limitations

Despite its strengths, the workflow has notable limitations. First, the dependency on pre-trained LLMs such as GPT-4o raises concerns about transparency and reproducibility, particularly in closed-source environments. Using open-source models avoids vendor lock-in, but they require ongoing updates and fine-tuning to stay relevant as clinical evidence evolves. Additionally, while Promptriever’s PEFT adaptation optimizes computational efficiency, it may still face challenges when addressing highly heterogenous datasets or rare conditions, which require more extensive contextual understanding. The retrieval process was designed to prioritize highly relevant studies, but in doing so, some potentially useful papers may have been excluded. The search parameters were structured to maximize precision over recall, ensuring that the selected studies were of high relevance. Future iterations could employ broader, multi-stage searches, ensuring a more comprehensive dataset while maintaining retrieval precision.

Moreover, the reliance on static pre-trained embeddings limits the pipeline’s real-time adaptability to newly emerging clinical evidence. This constraint is particularly evident in areas with rapidly evolving treatments, where retraining models may introduce delays. The robustness of Bayesian NMA outputs could also be impacted by the quality of initial inputs, necessitating rigorous pre-processing and curation. This work does not aim to establish or refine specific statistical models but rather to demonstrate the feasibility of an end-to-end automated pipeline. The focus is on proving that AI can successfully retrieve clinical studies, extract structured data, and execute NMAs with minimal human intervention. In this study, the *MetaMind* pipeline was primarily validated at the component level, with retrieval, data extraction, and network meta-analysis modelling evaluated separately to characterize performance and isolate sources of error. While this modular evaluation provides transparency and diagnostic insight, it does not fully capture the potential compounding effects of errors across stages in a fully end-to-end deployment. While the AI pipeline effectively automates these tasks, domain experts are still needed to validate statistical models, adjust assumptions, and interpret results in context. Further work should incorporate a wider search strategy and develop rule-based classifiers for eligibility screening, enabling the pipeline to both discover and autonomously investigate all potentially relevant trials. As a result, we did not conduct formal sensitivity analyses such as exclusion of studies at high risk of bias or systematic comparison of fixed- versus random-effects network meta-analysis models. Incorporating automated sensitivity analyses, including risk-of-bias–aware filtering and alternative model specifications, represents an important direction for future development of the *MetaMind* framework. Finally, while qualitative assessment of transitivity and network structure did not reveal major concerns, fully automated execution of these diagnostic procedures was not implemented in the current version of *MetaMind*.

The performance of *MetaMind* was evaluated on studies with sufficiently well-reported trial data, and we did not formally benchmark extraction or downstream impact for highly ambiguous or poorly reported studies (e.g., trials requiring imputation of missing standard deviations). While the system can flag such cases and suggest commonly used estimation approaches, the statistical validity of these suggestions and their influence on final effect estimates were not independently evaluated in this study. Assessing the robustness of automated assistance under conditions of incomplete or inconsistent reporting—particularly with respect to imputation choices and their downstream impact on treatment rankings—represents an important direction for future work.

## Future directions

To address these limitations, future research could explore the integration of agentic LLMs—autonomous AI agents capable of dynamically adapting to complex workflows [33]. Agentic LLMs could update models as new trial data become available, allowing quicker incorporation of emerging evidence into analyses. Systematic evaluation across open-source large language models would improve transparency and reproducibility, enabling benchmarking of retrieval, extraction, and aggregation performance beyond proprietary systems. Broader validation across additional therapeutic areas, outcome types, and study designs would strengthen generalizability claims. Tighter integration of structured risk-of-bias assessments and automated sensitivity analyses (e.g., exclusion of high-risk studies) would further enhance the reliability of downstream network meta-analytic conclusions. Finally, expanding the workflow to incorporate adaptive learning mechanisms and multilingual capabilities would make it more globally applicable, particularly in low-resource settings. By combining these advancements, the workflow could set new standards for automated clinical analytics, enabling broader accessibility and real-time adaptability in evidence synthesis tasks.

## Supporting information

S1 File Supplementary methods, figures, and tables. (DOCX)
