# Supplementary material for: MetaMind: A multi-agent transformer-driven framework for automated network meta-analyses
Source: PLoS One. 2026 Feb 13;21(2):e0342895. doi: 10.1371/journal.pone.0342895 (PMC12904386; doi:10.1371/journal.pone.0342895)
Supplement: S1 File — (DOCX) [file pone.0342895.s001.docx]

**Supplementary Material**

**Supplementary Figure 1: Promptriever Code**

import torch

import torch.nn.functional as F

from transformers import AutoTokenizer, AutoModel

from peft import PeftModel, PeftConfig

import numpy as np

from Bio import Entrez

class Promptriever:

    def __init__(self, model_name_or_path):

        self.model, self.tokenizer = self.get_model(model_name_or_path)

        self.model.eval().cuda()

    def get_model(self, peft_model_name):

        # Load the PEFT configuration to get the base model name

        peft_config = PeftConfig.from_pretrained(peft_model_name)

        base_model_name = peft_config.base_model_name_or_path

        # Load the base model and tokenizer

        base_model = AutoModel.from_pretrained(base_model_name)

        tokenizer = AutoTokenizer.from_pretrained(base_model_name)

        tokenizer.pad_token = tokenizer.eos_token

        tokenizer.pad_token_id = tokenizer.eos_token_id

        tokenizer.padding_side = "right"

        # Load and merge the PEFT model

        model = PeftModel.from_pretrained(base_model, peft_model_name)

        model = model.merge_and_unload()

        # Configure max length for the model

        model.config.max_length = 512

        tokenizer.model_max_length = 512

        return model, tokenizer

    def create_batch_dict(self, tokenizer, input_texts):

        max_length = self.model.config.max_length

        batch_dict = tokenizer(

            input_texts,

            max_length=max_length - 1,

            return_token_type_ids=False,

            return_attention_mask=False,

            padding=False,

            truncation=True,

        )

        batch_dict["input_ids"] = [

            input_ids + [tokenizer.eos_token_id]

            for input_ids in batch_dict["input_ids"]

        ]

        return tokenizer.pad(

            batch_dict,

            padding=True,

            pad_to_multiple_of=8,

            return_attention_mask=True,

            return_tensors="pt",

        )

    def encode(self, sentences, max_length: int = 2048, batch_size: int = 4):

        all_embeddings = []

        for i in range(0, len(sentences), batch_size):

            batch_texts = sentences[i : i + batch_size]

            batch_dict = self.create_batch_dict(self.tokenizer, batch_texts)

            batch_dict = {

                key: value.to(self.model.device) for key, value in batch_dict.items()

            }

            with torch.cuda.amp.autocast():

                with torch.no_grad():

                    outputs = self.model(**batch_dict)

                    last_hidden_state = outputs.last_hidden_state

                    sequence_lengths = batch_dict["attention_mask"].sum(dim=1) - 1

                    batch_size = last_hidden_state.shape[0]

                    reps = last_hidden_state[

                        torch.arange(batch_size, device=last_hidden_state.device),

                        sequence_lengths,

                    ]

                    embeddings = F.normalize(reps, p=2, dim=-1)

                    all_embeddings.append(embeddings.cpu().numpy())

        return np.concatenate(all_embeddings, axis=0)

# PubMed Search Function

def search_pubmed(query, max_results=100):

    Entrez.email = "achilleas.livieratos@gmail.com"  # Add your email here

    handle = Entrez.esearch(

        db="pubmed",

        term=query,

        retmax=max_results,

        retmode="xml",

        sort="relevance",

    )

    results = Entrez.read(handle)

    handle.close()

    return results["IdList"]

# Initialize the Promptriever model

model = Promptriever("samaya-ai/promptriever-llama2-7b-v1")

# PubMed query

query = (

    "ulcerative colitis AND placebo AND (mirikizumab OR upadacitinib OR filgotinib OR ustekinumab OR tofacitinib OR etrasimod)"

)

# Fetch PubMed IDs

pubmed_ids = search_pubmed(query, max_results=100)

print("PubMed IDs retrieved:", pubmed_ids)

if pubmed_ids:

    # Fetch articles from PubMed

    handle = Entrez.efetch(db="pubmed", id=pubmed_ids, retmode="xml")

    records = Entrez.read(handle)

    handle.close()

    # Extract abstracts and metadata

    documents = []

    metadata = []

    for article in records["PubmedArticle"]:

        title = article["MedlineCitation"]["Article"].get("ArticleTitle", "No Title")

        journal = article["MedlineCitation"]["Article"]["Journal"].get("Title", "No Journal")

        pub_date = article["MedlineCitation"]["Article"]["Journal"].get("JournalIssue", {}).get("PubDate", "No Date")

        abstract = article["MedlineCitation"]["Article"].get("Abstract", {}).get("AbstractText", [])

        if abstract:

            documents.append(" ".join(abstract))

            metadata.append({"Title": title, "Journal": journal, "PubDate": pub_date})

    # Specify target journals

    TARGET_JOURNALS = {"Lancet (London, England)", "The New England journal of medicine"}

    # Filter documents for specific journals

    filtered_documents = []

    filtered_metadata = []

    for idx, meta in enumerate(metadata):

        if meta["Journal"] in TARGET_JOURNALS:

            filtered_documents.append(documents[idx])

            filtered_metadata.append(meta)

    # Check if filtered results are available

    if not filtered_documents:

        print("No relevant articles found in the specified journals.")

    else:

        # Define query instruction

        instruction = (

            "A relevant document would describe clinical trials on ulcerative colitis "

            "where patients were tested against placebo. The document should detail the use of treatments like "

            "mirikizumab, upadacitinib, filgotinib, or ustekinumab or etrasimod or tofacitinib and include clinical efficacy results."

        )

        input_text = f"query: {query.strip()} {instruction.strip()}".strip()

        # Encode query and the filtered documents

        query_embedding = model.encode([input_text])

        doc_embeddings = model.encode(filtered_documents)

        # Calculate similarities

        similarities = np.dot(query_embedding, doc_embeddings.T)[0]

        sorted_indices = np.argsort(similarities)[::-1]

        # Display top results

        print("Top relevant PubMed articles in specified journals:")

        for idx in sorted_indices[:10]:  # Top 10 results

            print(f"Title: {filtered_metadata[idx]['Title']}")

            print(f"Journal: {filtered_metadata[idx]['Journal']}")

            print(f"Publication Date: {filtered_metadata[idx]['PubDate']}")

            print(f"Abstract: {filtered_documents[idx]}")

            print(f"Similarity: {similarities[idx]:.4f}\n")

else:

    print("No PubMed articles found.")

**Supplementary Figure 2: Mixture of Agents Code**

import numpy as np

import fitz  # PyMuPDF for extracting text from PDFs

import aiohttp

import asyncio

import openai

import nest_asyncio

# Apply nest_asyncio to allow nested event loops

nest_asyncio.apply()

# Set your API keys

together_api_key = "Your Key"

openai.api_key = "Your Key"

# Together AI base URL for model inference

together_api_url = "https://api.together.xyz/v1/completions"

headers = {

    "Authorization": f"Bearer {together_api_key}",

    "Content-Type": "application/json"

}

# Generate model-specific prompts for clinical data extraction

def get_model_prompts(abstract_text):

    return {

        "meta-llama/Meta-Llama-3.1-8B-Instruct-Turbo": f"""

        Extract detailed clinical data from the text below:

        {abstract_text}

        Provide the following information:

        - Treatment names and endpoints.

        - Baseline clinical values (Mean, SD, or CI) and explicitly state if CI or SD is missing.

        - Final clinical remission values, including calculated Mean, SDs, or CIs, and explicitly indicate any missing values.

        - Weekly remission percentages (Weeks 4, 8, and 12) with SDs or CIs for all treatment groups.

        - Sample sizes for each treatment group.

        If any SD or CI values are missing, highlight this explicitly and suggest plausible methods for estimating them.

        Format the output as a structured table suitable for Network Meta-Analysis (NMA).

        """,

        "mistralai/Mistral-7B-Instruct-v0.3": f"""

        Extract clinical data for induction phase from the following text:

        {abstract_text}

        Include:

        - Treatment names and endpoints.

        - Baseline and final remission values with SDs or CIs, clearly identifying missing data.

        - Weekly remission percentages (Weeks 4, 8, and 12) with SDs or CIs for all treatment groups.

        - Sample sizes for each treatment group.

        For any missing SD or CI values, indicate their absence explicitly and propose how they might be calculated.

        Provide results in a structured table for NMA.

        """,

        "Qwen/Qwen2-72B-Instruct": f"""

        Extract induction phase clinical data from the following text:

        {abstract_text}

        Provide:

        - Baseline clinical values (Mean, SD, or CI) and clearly indicate if these values are missing.

        - Final clinical remission values with calculated Mean, SDs, or CIs, and highlight any missing values.

        - Weekly remission percentages (Weeks 4, 8, and 12) with SDs or CIs for all treatment groups.

        - Sample sizes for all treatment groups.

        Emphasize identifying and reporting missing SD or CI values, and suggest estimation methods if possible.

        Summarize in a structured format suitable for NMA.

        """

    }

# Aggregator prompt template

aggregator_prompt_template = """

    You are provided with multiple responses from different models analyzing clinical study data.

    Your task is to extract the following data for a Network Meta-Analysis (NMA):

    - Baseline clinical values for each treatment group (Mean, SD, or CI). Indicate if CI or SD values are missing.

    - Final clinical remission values for each treatment group, with calculated Mean, SDs, or CIs, and explicitly flag missing values.

    - Weekly remission percentages (Weeks 4, 8, 12) for each treatment group with SDs or CIs.

    - Sample sizes for all treatment groups.

    Highlight any missing SD or CI values and propose methods for estimation if feasible.

    Summarize the output in a clear, tabular format suitable for NMA.

"""

# Helper function to validate and log missing CI/SD values

def validate_ci_sd_extraction(batch_result):

    missing_data_flag = False

    if "CI" not in batch_result and "SD" not in batch_result:

        missing_data_flag = True

    return missing_data_flag, batch_result

async def run_together_model_with_retry(model, prompt):

    payload = {"model": model, "prompt": prompt, "max_tokens": 2048}

    async with aiohttp.ClientSession() as session:

        for _ in range(3):  # Retry logic

            async with session.post(together_api_url, json=payload, headers=headers) as response:

                if response.status == 200:

                    return await response.text()

    return None  # Return None if all retries fail

# Helper function to synthesize responses with GPT-4o

async def run_llm(model, prompt, prev_responses=None):

    system_prompt = "You are provided with multiple responses to a user query. Please synthesize these responses into coherent, accurate responses and calculate Mean clinical values, remission percentages, and Standard deviations (SDs) or confidence intervals (CIs) for each treatment group."

    try:

        if prev_responses:

            combined_responses = "\n\n".join(prev_responses)

            prompt = f"{system_prompt}\n\nPrevious responses:\n{combined_responses}"

        if model == "gpt-4o":

            # Await the coroutine to get the response object

            response = await openai.ChatCompletion.acreate(

                model="gpt-4o",

                messages=[

                    {"role": "system", "content": system_prompt},

                    {"role": "user", "content": prompt}

                ],

                max_tokens=2048,

                temperature=0.7,

                stream=True

            )

            # Collect the streamed output into a single string

            response_content = ""

            async for chunk in response:  # Iterate over the response object

                if "choices" in chunk:

                    response_content += chunk["choices"][0]["delta"].get("content", "")

            return response_content.strip()  # Return the full response as a string

        else:

            return await run_together_model_with_retry(model, prompt)

    except Exception as e:

        print(f"Error with model {model}: {e}")

        return None

def extract_text_from_pdfs_in_batches(pdf_path):

    with fitz.open(pdf_path) as pdf:

        # Extract all text from the PDF

        full_text = ""

        for page in pdf:

            full_text += page.get_text()

        # Split text into two roughly equal parts

        midpoint = len(full_text) // 2

        return [full_text[:midpoint], full_text[midpoint:]]

# Multi-layer aggregation with batch-level and overall streaming

async def process_pdf_batches(pdf_path):

    text_batches = extract_text_from_pdfs_in_batches(pdf_path)

    final_aggregated_data = []

    for i, batch_text in enumerate(text_batches):

        print(f"\nProcessing Batch {i + 1}/{len(text_batches)}...\n")

        model_prompts = get_model_prompts(batch_text)

        # Layer 1: Run reference models asynchronously

        initial_responses = await asyncio.gather(

            *[run_llm(model, model_prompts[model]) for model in model_prompts]

        )

        # Filter out None responses

        valid_responses = [r for r in initial_responses if r]

        # Layers 2 and 3: Iterative aggregation with GPT-4o

        for layer in range(2):

            print(f"\nRunning Aggregation Layer {layer + 2}...\n")

            valid_responses = await asyncio.gather(

                *[run_llm("gpt-4o", aggregator_prompt_template, prev_responses=valid_responses) for _ in model_prompts]

            )

            valid_responses = [r for r in valid_responses if r]  # Filter out None again

        # Validate for CI/SD presence and highlight missing data

        for response in valid_responses:

            missing_ci_sd, validated_result = validate_ci_sd_extraction(response)

            if missing_ci_sd:

                print(f"Warning: Missing CI/SD in Batch {i + 1}: {validated_result}\n")

        # Store aggregated batch result

        if valid_responses:

            batch_result = "\n".join(valid_responses)

            print(f"\nDetailed Batch {i + 1} Output:\n{batch_result}\n")

            final_aggregated_data.append(batch_result)

        else:

            print(f"Batch {i + 1} failed to produce valid responses.")

    # Combine all batch results for overall aggregation

    if final_aggregated_data:

        combined_final_prompt = "\n\n".join(final_aggregated_data)

        print("\n================== Final Document Aggregated Output ==================\n")

        final_stream = await openai.ChatCompletion.acreate(

            model="gpt-4o",

            messages=[

                {"role": "system", "content": aggregator_prompt_template},

                {"role": "user", "content": combined_final_prompt}

            ],

            max_tokens=2048,

            temperature=0.7,

            stream=True

        )

        # Stream overall output

        final_output = ""

        async for chunk in final_stream:

            if "choices" in chunk:

                final_output += chunk["choices"][0]["delta"].get("content", "")

                print(chunk["choices"][0]["delta"].get("content", ""), end='', flush=True)

        return final_output.strip()

# Main async function

async def main():

    pdf_path = "/content/adalimumab.pdf"  # Update with the actual path

    await process_pdf_batches(pdf_path)

# Run the script

await main()

**Supplementary Figure 3: FLASK UC and CD Plots**


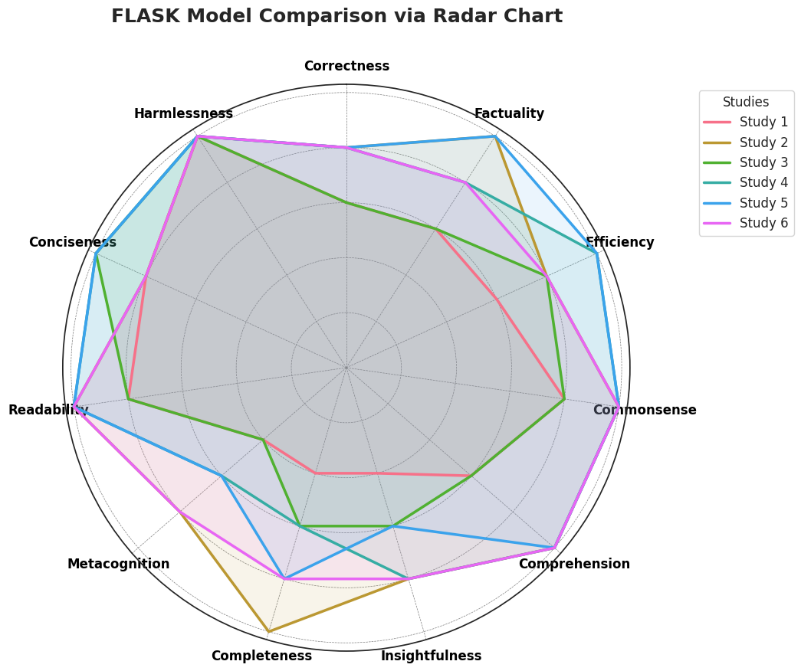

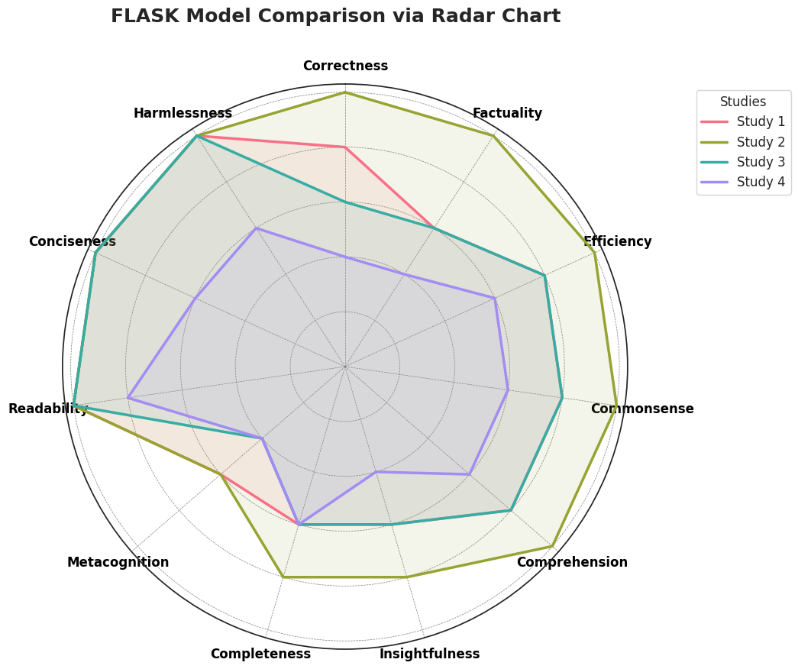


Variability in FLASK metrics is associated with broad prompts not always applicable for each publication.

**Supplementary Figure 4: Code-NMA Script Generation**

import requests

import os

import subprocess

import re

API_URL = "https://api.openai.com/v1/chat/completions"

API_KEY = "Your Key"

# Pseudocode instructions for OpenAI to generate the R script

prompt_instructions = """

Generate an R script that follows these pseudocode instructions exactly:

Load necessary libraries: brms, dplyr, tibble.

Define a dataset (`UC_data`) with:

   - Study details (study_id).

   - Treatment groups (treatment).

   - Response counts (response_count).

   - Total sample sizes (total_sample_size).

Ensure placebo is the reference category for treatment comparisons.

```r

UC_data$treatment <- relevel(factor(UC_data$treatment), ref = "placebo")```

Fit a Bayesian random-effects model using:

   - **Binomial likelihood (logit link) for response counts over total sample size.**

   - **Fixed effects:** Treatment group comparisons (**placebo as reference**).

   - **Random effects:** Study-level variability, allowing **random intercepts and slopes** for treatment.

   - **Formula:**

     ```

     response_count | trials(total_sample_size) ~ treatment + (1 + treatment | study_id)

     ```

   - **Priors:**

     - Treatment effects: `Normal(0, 3.5)`

     - Study variance: `Normal(0, 0.5)`, constrained to non-negative values (`lb=0`).

   - **Sampling settings:**

     - `chains = 4`, `iter = 2000`, `warmup = 1000`, `seed = 123`, `adapt_delta = 0.98`

 Extracts posterior summaries:

       - Computes mean estimates, standard errors, and 95% credible intervals (2.5%, 50%, 97.5% quantiles) using:

         ```r

         posterior_summ <- as.data.frame(posterior_summary(model, probs = c(0.025, 0.5, 0.975)))

         ```

   - Extracts diagnostics:

       - Obtains summary of the fitted model:

         ```r

         model_summary <- summary(model)

         diagnostics <- as.data.frame(model_summary$fixed)

         ```

   - Converts row names to a column to facilitate merging of Rhat values:

       ```r

       posterior_summ <- posterior_summ %>% rownames_to_column("Parameter")

       diagnostics <- diagnostics %>% rownames_to_column("Parameter") %>% select(Parameter, Rhat)

       ```

   - Renames parameters for consistency by removing the `b_` prefix:

       ```r

       posterior_summ$Parameter <- gsub("^b_", "", posterior_summ$Parameter)

       ```

   - Merges posterior summaries with Rhat values:

       ```r

       posterior_summ_df <- left_join(posterior_summ, diagnostics, by = "Parameter") %>%

         filter(grepl("^treatment", Parameter)) %>%

         mutate(Parameter = gsub("^treatment", "d", Parameter)) %>%

         select(Parameter, Estimate, Est.Error, Q2.5, Q97.5, Rhat)

       ```

   - Prints the final posterior summary table:

       ```r

       cat("\nFinal Posterior Summary with Rhat Values:\n")

       print(posterior_summ_df)

       ```

   - Extracts and prints the between-study heterogeneity estimate (tau):

       ```r

       tau_value <- as.numeric(VarCorr(model)$study_id$sd[1])

       cat("\nTau (between-study heterogeneity):", tau_value, "\n")

       ```

   - Displays model diagnostics:

       ```r

       cat("\nSamples were drawn using NUTS(diag_e) at", Sys.time(), "\n")

       cat("For each parameter, n_eff is a crude measure of effective sample size,\n")

       cat("and Rhat is the potential scale reduction factor on split chains (at\n")

       cat("convergence, Rhat=1).\n")

       ```

2. Execute the R script inline using Python:

   - Use `subprocess.run()` to execute the R script using `Rscript -e`.

   - Capture both standard output and errors to verify correct execution.

   - Print all relevant outputs for user inspection.

3. Expected output:

   - A summary table displaying treatment effect estimates, confidence intervals, and Rhat values.

   - The between-study heterogeneity estimate (tau).

   - Convergence diagnostics including effective sample size and Rhat values.

Ensure the LLM generates the entire R script based on these instructions without explicitly providing any R code.

"""

# Construct request payload

payload = {

    "model": "gpt-4o",

    "messages": [

        {"role": "system", "content": "You are a helpful assistant that generates R scripts based on given pseudocode."},

        {"role": "user", "content": prompt_instructions}

    ],

    "max_tokens": 3000,

    "temperature": 0.0

}

# Send API request to OpenAI

headers = {

    "Authorization": f"Bearer {API_KEY}",

    "Content-Type": "application/json"

}

response = requests.post(API_URL, json=payload, headers=headers)

# Handle response

if response.status_code == 200:

    response_data = response.json()

    r_script = response_data.get("choices", [{}])[0].get("message", {}).get("content", "").strip()

    if r_script:

        print("Generated R Script:\n")

        print(r_script)

        # Execute the R script using subprocess

        print("\nExecuting the R script...\n")

        result = subprocess.run(["Rscript", "-e", r_script], capture_output=True, text=True)

        # Display the output

        print("Standard Output:\n", result.stdout)

        print("Standard Error:\n", result.stderr)

    else:

        print("No R script was returned. Check the API response.")

else:

    print(f"Error {response.status_code}: {response.text}")

**Supplementary Figure 5: UC and CD Manual NMA Validation**

| **UC Therapeutic Intervention** | **Estimate** | **Est. Error** | **Q2.5** | **Q97.5** | **Rhat** |
| --- | --- | --- | --- | --- | --- |
| Etrasimod | 1.09 | 0.62 | -0.14 | 2.4 | 1.0 |
| Filgotinib100mg | 0.44 | 0.81 | -1.29 | 2.15 | 1.0 |
| Filgotinib200mg | 0.76 | 0.84 | -1.07 | 2.63 | 1.0 |
| Mirikizumab | 0.73 | 0.78 | -1.01 | 2.38 | 1.0 |
| Tofacitinib | 1.3 | 0.64 | 0.06 | 2.73 | 1.0 |
| Upadacitinib | 2.3 | 0.6 | 1.13 | 3.53 | 1.0 |
| Ustekinumab 6mg/kg | 1.16 | 0.85 | -0.56 | 2.91 | 1.0 |
| Ustekinumab130mg | 1.21 | 0.88 | -0.61 | 3.02 | 1.0 |

Tau (between-study heterogeneity): 0.66

| **CD Therapeutic Intervention** | **Estimate** | **Est. Error** | **Q2.5** | **Q97.5** | **Rhat** |
| --- | --- | --- | --- | --- | --- |
| Filgotinib 200mg QD | 1.15 | 0.49 | 0.22 | 2.1 | 1.0 |
| Risankizumab 200mg | 0.58 | 0.69 | -0.74 | 2.01 | 1.0 |
| Risankizumab 600mg | 1.19 | 0.65 | -0.09 | 2.46 | 1.0 |
| Upadacitnib 45 mg | 0.87 | 0.26 | 0.33 | 1.46 | 1.01 |
| Ustekinumab 6mg/kg | 1.11 | 0.27 | 0.62 | 1.71 | 1.01 |
| Ustekinumab 130mg | 0.72 | 0.34 | 0.12 | 1.32 | 1.02 |

Tau (between-study heterogeneity): 0.22

**Supplementary Figure 6: UC and CD Network Plots**

**
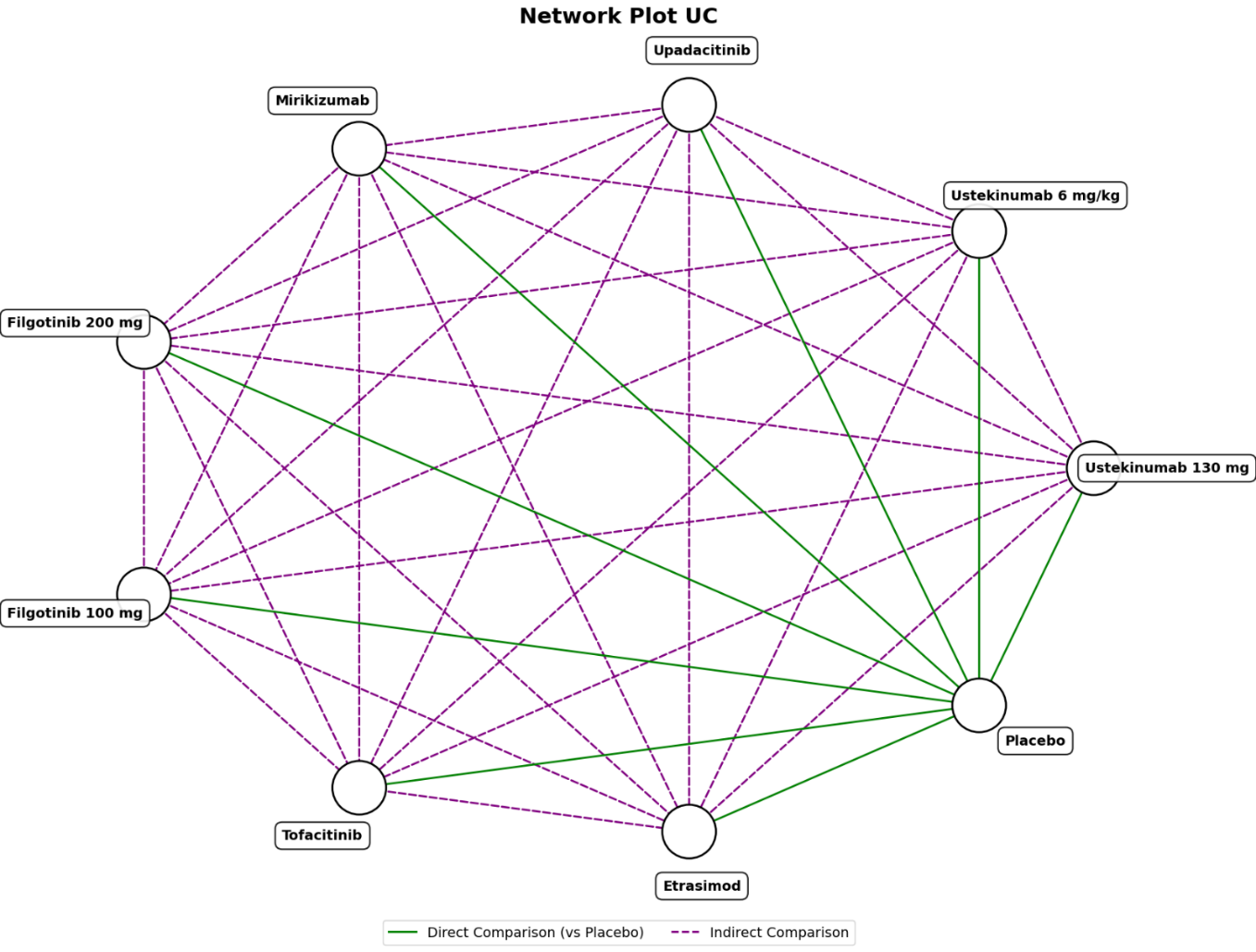
**

**
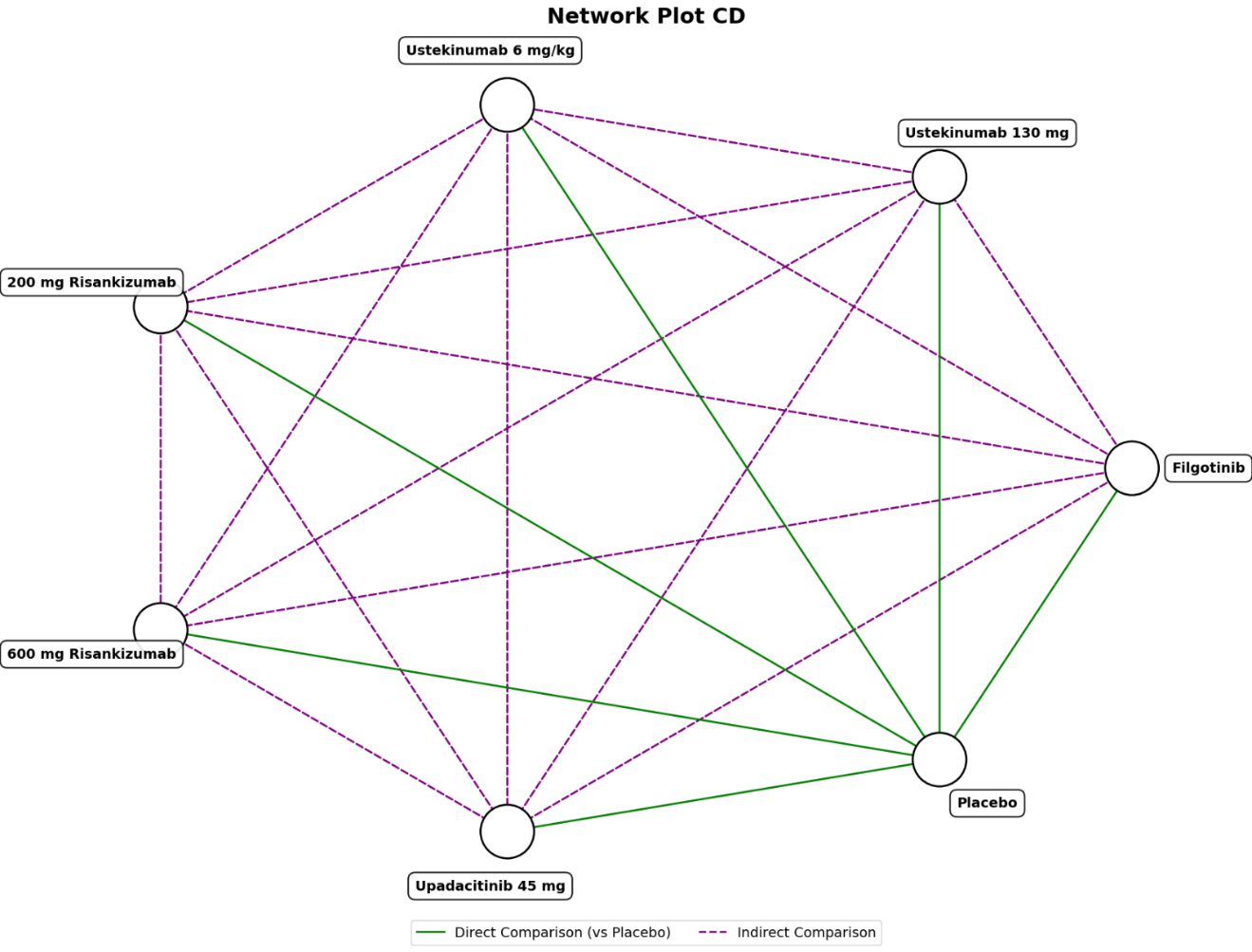
**

**Supplementary Figure 7: Information Retrieval using Promptriever versus Published NMA**


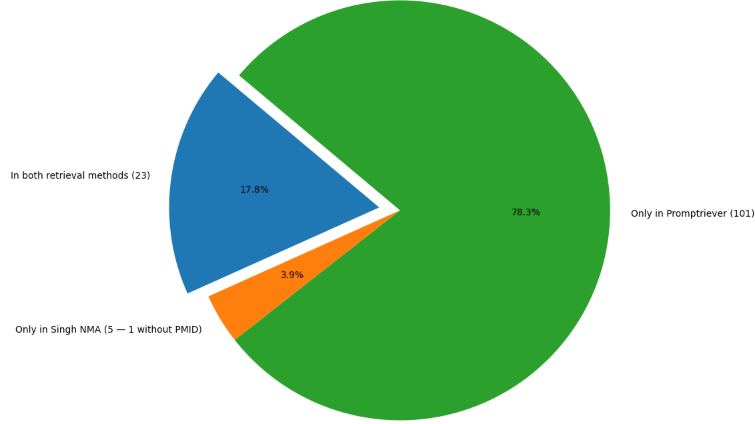


Promptriever recovered the vast majority (78.3%) of studies not captured by Singh et al.’s manual NMA—many of which were recent PubMed-indexed trials or combination-therapy investigations that post-dated the original review. Only 3.9% of trials were uniquely in the published NMA (and 1 study not PubMed-indexed), while 17.8% appeared in both approaches. This suggests that Promptriever reproduces nearly all of the published NMA’s PubMed-sourced evidence and identifies additional recent and diverse treatment studies.

**Supplementary Figure 8: NMA Generation using *Metamind***


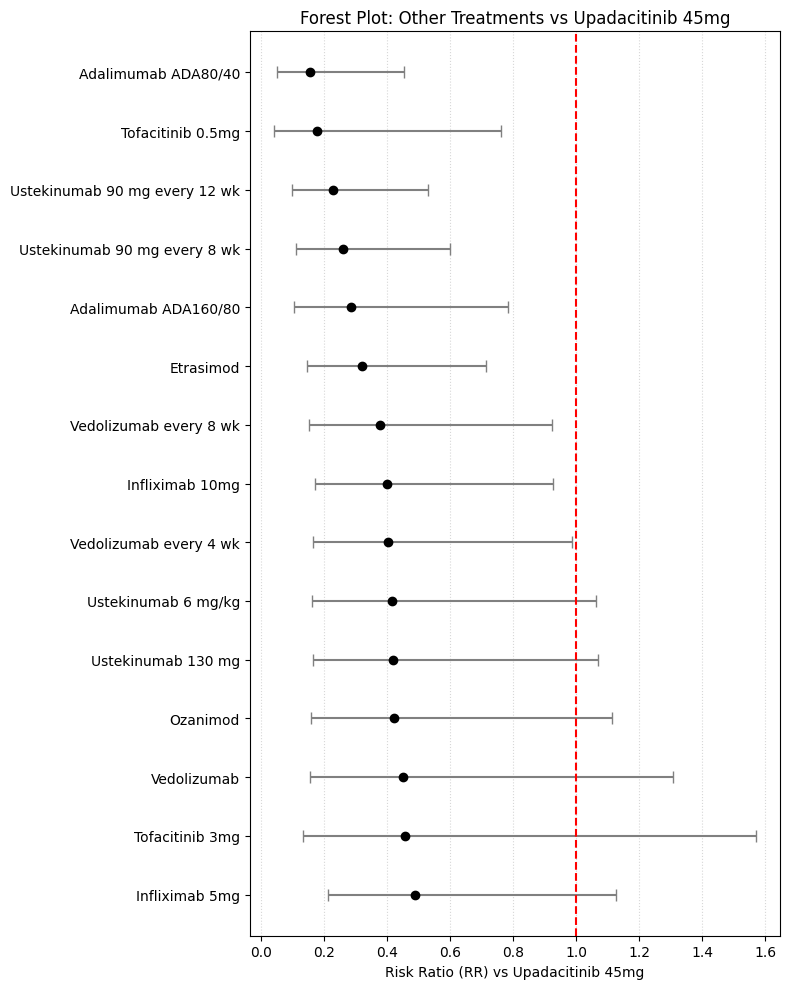


Note: NMA generated from a total of 8 RCT studies

**Supplementary Figure 9: Code-NMA Script Execution**

Generated R Script:

Below is the R script generated based on the provided pseudocode instructions:

```r

# Load necessary libraries

library(brms)

library(dplyr)

library(tibble)

# Define the dataset

UC_data <- tibble(

study_id = c(1, 2, 3), # Example study IDs

treatment = c("placebo", "treatment1", "treatment2"), # Example treatments

response_count = c(10, 15, 20), # Example response counts

total_sample_size = c(100, 150, 200) # Example total sample sizes

)

# Ensure placebo is the reference category for treatment comparisons

UC_data$treatment <- relevel(factor(UC_data$treatment), ref = "placebo")

# Fit a Bayesian random-effects model

model <- brm(

formula = response_count | trials(total_sample_size) ~ treatment + (1 + treatment | study_id),

data = UC_data,

family = binomial(link = "logit"),

prior = c(

set_prior("normal(0, 3.5)", class = "b"),

set_prior("normal(0, 0.5)", class = "sd", lb = 0)

),

chains = 4,

iter = 2000,

warmup = 1000,

seed = 123,

control = list(adapt_delta = 0.98)

)

# Extract posterior summaries

posterior_summ <- as.data.frame(posterior_summary(model, probs = c(0.025, 0.5, 0.975)))

# Extract diagnostics

model_summary <- summary(model)

diagnostics <- as.data.frame(model_summary$fixed)

# Convert row names to a column

posterior_summ <- posterior_summ %>% rownames_to_column("Parameter")

diagnostics <- diagnostics %>% rownames_to_column("Parameter") %>% select(Parameter, Rhat)

# Rename parameters for consistency

posterior_summ$Parameter <- gsub("^b_", "", posterior_summ$Parameter)

# Merge posterior summaries with Rhat values

posterior_summ_df <- left_join(posterior_summ, diagnostics, by = "Parameter") %>%

filter(grepl("^treatment", Parameter)) %>%

mutate(Parameter = gsub("^treatment", "d", Parameter)) %>%

select(Parameter, Estimate, Est.Error, Q2.5, Q97.5, Rhat)

# Print the final posterior summary table

cat("

Final Posterior Summary with Rhat Values:

")

print(posterior_summ_df)

# Extract and print the between-study heterogeneity estimate (tau)

tau_value <- as.numeric(VarCorr(model)$study_id$sd[1])

cat("

Tau (between-study heterogeneity):", tau_value, "

")

# Display model diagnostics

cat("

Samples were drawn using NUTS(diag_e) at", Sys.time(), "

")

cat("For each parameter, n_eff is a crude measure of effective sample size,

")

cat("and Rhat is the potential scale reduction factor on split chains (at

")

cat("convergence, Rhat=1).

")

```

To execute this R script inline using Python, you can use the `subprocess` module. Here is an example of how you might do this:

```python

import subprocess

# Define the R script as a string

r_script = """

# (Insert the R script here)

"""

# Execute the R script using subprocess

result = subprocess.run(

["Rscript", "-e", r_script],

capture_output=True,

text=True

)

# Print the standard output and errors

print("Standard Output:\n", result.stdout)

print("Standard Error:\n", result.stderr)

```
